# Beta-band network modularity in resting-state EEG negatively correlates with level of intelligence

**DOI:** 10.1192/j.eurpsy.2022.1639

**Published:** 2022-09-01

**Authors:** A. Komarova, E. Shcherbakova, A. Kiselnikov, D. Mitiureva, M. Yurlova, P. Kabanova, E. Slovenko, E. Terlichenko, I. Tan, V. Zubko

**Affiliations:** Lomonosov Moscow State University, Psychology, Moscow, Russian Federation

**Keywords:** Modularity, Intelligence, Resting-state EEG

## Abstract

**Introduction:**

Recent studies mostly focus on the links between measures of alpha-band EEG networks and intelligence. However, associations between wide frequency range EEG networks and general intelligence level remain underresearched.

**Objectives:**

In this study in a student sample we aimed to correlate the intelligence level and graph metrics of the sensors/sources-level networks constructed in different frequency EEG bands.

**Methods:**

We recorded eyes-closed resting-state EEG in 28 healthy participants (21.4±2.1 y.o., 18 females, 1 left-handed). The Raven’s Standard Progressive Matrices Plus (‘SPM Plus’, 60 figures) was used as an intelligence measure. We constructed networks for all possible combinations of sensors/sources-level and 4-8, 8-13, 13-30, or 4-30 Hz frequency bands using Weighted Phase-Lag Index (wPLI), and calculated four graph metrics (Characteristic Path Length, Clustering Coefficient, Modularity, and Small World Index) for each network. Spearman correlation (with Holm-Sidak correction) was applied to characterize the relations between the SPM Plus scores and all the network metrics.

**Results:**

SPM Plus scores varied from 35 to 57 (mean 45.3±4.2), and the intelligence level negatively correlated with Modularity in beta-band (r = -0.63, p_corr_ = 0.0253).

**Conclusions:**

High modularity may reflect relatively high segregation, but not integration, of networks (Girn, Mills, Christoff, 2019). Accordingly, our findings may shed light on the neural mechanisms of the general inefficiency of global cognitive processing in the case of intellectual decline related to different mental disorders. *Funding: This research has been supported by the Interdisciplinary Scientific and Educational School of Lomonosov Moscow State University ‘Brain, Cognitive Systems, Artificial Intelligence’.*

**Disclosure:**

No significant relationships.

